# Neurophysiological Correlates of Fast Mapping of Novel Words in the Adult Brain

**DOI:** 10.3389/fnhum.2019.00304

**Published:** 2019-09-19

**Authors:** Marina J. Vasilyeva, Veronika M. Knyazeva, Aleksander A. Aleksandrov, Yury Shtyrov

**Affiliations:** ^1^Department of Higher Nervous Activity and Psychophysiology, Saint Petersburg State University, Saint Petersburg, Russia; ^2^Laboratory of Behavioral Neurodynamics, Saint Petersburg State University, Saint Petersburg, Russia; ^3^Center of Functionally Integrative Neuroscience, Aarhus University, Aarhus, Denmark

**Keywords:** brain, event-related potentials, language, fast mapping, word, semantic, learning, acquisition

## Abstract

Word acquisition could be mediated by the neurocognitive mechanism known as fast mapping (FM). It refers to a process of incidental exclusion-based learning and is believed to be a critical mechanism for the rapid build-up of lexicon, although its neural mechanisms are still poorly understood. To investigate the neural bases of this key learning skill, we used event-related potentials (ERPs) and employed an audio-visual paradigm that included a counterbalanced set of familiar and novel spoken word forms presented, in a single exposure, in conjunction with novel and familiar images. To define learning-related brain dynamics, passive auditory ERPs, known to index long-term memory trace activation, were recorded before and after the FM task. Following the single FM learning exposure, we found a significant enhancement in neural activation elicited by the newly trained word form, which was expressed at ~200–400 ms after the word onset. No similar amplitude increase was found either for the native familiar word used as a control stimulus in the same learning paradigm or for similar control stimuli which were not subject to training. Topographic analysis suggested a left-lateral shift of the ERP scalp distribution for the novel FM word form, underpinned by fronto-temporal cortical sources, which may indicate the involvement of pre-existing neurolinguistic networks for mastering new word forms with native phonology. Overall, the near-instant changes in neural activity after a single-shot novel word training indicate that FM could promote rapid integration of newly learned items into the brain’s neural lexicon, even in adulthood.

## Introduction

From birth and throughout their entire lives, human beings learn vast amounts of new and diverse information. This is especially true in the domain of language where incredibly rapid word learning processes enable efficient mother tongue acquisition during childhood as well as mastering a second language or professional lexicon later in life. Despite numerous studies, identification of distinct neurobiological indices and putative mechanisms of such rapid word learning remains challenging.

A large number of studies have posited that rapid new word acquisition could be mediated by the neurocognitive mechanism known as *fast mapping* (FM). FM refers to a process of incidental exclusion-based learning which promotes rapid integration of newly learned items into cortical memory networks (Sharon et al., 2011; Coutanche and Thompson-Schill, 2014; Atir-Sharon et al., 2015). It was first described by Carey and Bartlett (1978) in their seminal “chromium study” with young children. These authors characterized FM as a cognitive mechanism whereby, under conditions of experimentally created ambiguity, one can infer the meaning of a new word on the principle of mutual exclusivity, and the memory trace of such newly formed associations are established and maintained even after a single exposure to the novel item. The authors noted that, for a successful mapping, a child must be able to perform “referent selection” and “referent retention” corresponding to the newly learned word. In their experiment, Carey and Bartlett presented 3–4 year-old children with two trays, one of which was red and the other one olive, and asked to bring them the “chromium” tray. Children brought the olive tray, concluding that the new word “chromium” refers to this previously unknown color. In addition, one week later, children successfully chose the “chromium” tray among six different trays, demonstrating long-term retention of the representation of the newly learned word. Further studies revealed that young children are able to reproduce new words even one month after their single presentation (Markson and Bloom, 1997; Kalashnikova et al., 2014; but see Horst and Samuelson, 2008) and that with maturation the FM mechanism becomes even more efficient.

It is now assumed that FM is a critical mechanism serving the rapid build-up of lexicon, particularly at early stages of language acquisition. Since the pioneering work of Carey and Bartlett (1978), FM has been investigated quite extensively and has been described not only in humans but also in other mammals [for example, in primates (Cook and Fagot, 2009), dogs (Kaminski et al., 2004; Pilley and Reid, 2011)] and even in birds (Cook and Fagot, 2009). However, despite numerous studies, a considerable amount of contradictory results is still evident. Moreover, only few studies evaluated FM in adults, using predominantly behavioral measures. Finally, the exact neural underpinnings of this trait remain poorly understood, as neurophysiological research in this field has been limited.

Sharon et al. (2011) reported normal learning of new word forms under FM procedure in four middle-aged patients with anterograde amnesia following hippocampal damage: their memory performance did not differ from that in healthy matched controls not only after a 10 min delay, but also one week later, whereas under a standard explicit encoding (EE) condition (i.e., not inference-based direct instruction) these individuals showed impaired explicit memory at both delays. On the other hand, patients with anterior temporal lobe (ATL) lesions (but intact hippocampi) revealed no advantage for FM condition. These and other similar results suggested FM as a hippocampally-independent learning mechanism promoting rapid neocortically-based memory formation. However, other studies in patients with hippocampal injury using slightly modified paradigms failed to replicate FM benefits over the EE procedure (Smith et al., 2014; Warren and Duff, 2014; Warren et al., 2016).

In spite of these contradictory patient studies, recent findings in healthy young adults confirmed that learning through FM may accelerate rapid integration of newly learned items into cortical memory networks (Coutanche and Thompson-Schill, 2014), with an implicit memory measure (reaction time in a lexical task, applied after a 10-min delay as well as on the following day) revealing strong lexical competition following the FM learning procedure, while no similar evidence of lexical integration was found for EE condition. The authors proposed this pattern—rapid lexical integration of newly learned items manifest to a greater extent after incidental learning—to be a behavioral signature of FM (Coutanche and Thompson-Schill, 2014; Coutanche and Koch, 2017).

Several neuroimaging studies have claimed that FM may be linked to distinct neuroanatomical substrates. In one study the retrieval of semantic associations acquired through FM and EE conditions by healthy young subjects was measured during four alternative forced-choice recognition test using BOLD-fMRI (Merhav et al., 2015). Results indicated a specifically increased activity in ATL during retrieval in FM. Moreover, whereas a typical overnight strengthening of vmPFC engagement and vmPFC-hippocampal-neocortical interactions were apparent for EE, reflecting slower-rate consolidation processes, no similar increase was found for FM learning. The authors concluded that associative semantic learning through FM could be supported by the ATL as a critical hub enabling direct neocortical learning, bypassing the hippocampus and consolidation stage. This and other studies (e.g., Atir-Sharon et al., 2015) emphasize FM as a key neurocognitive mechanism enabling rapid neocortical plasticity to create novel semantic representations, largely supported by the temporal lobe without or minimal hippocampal involvement.

Such studies, therefore, propose a hippocampally-independent route of rapid cortical learning, without the slower consolidation stage. This somewhat contradicts the mainstream memory theories postulating that the initial fast stages of learning are hippocampus based while the neocortical memory systems are slower and require at least an overnight consolidation stage to form new representations (McClelland et al., 1995). In their seminal work, Davis and Gaskel (2009) applied principles from the Complementary Learning Systems (CLS; McClelland et al., 1995; McClelland, 2013) model of memory to brain mechanisms of word learning. According to this complementary systems account of word learning, there are two stages of lexical acquisition: (i) rapid initial encoding, largely supported by medial temporal lobe (MTL) and hippocampus, that is followed by (ii) slow lexical consolidation achieved offline in neocortex. Generally, this framework suggests that complementary systems in the hippocampus and neocortex maintain the ability to acquire new words and integrate them with existing linguistic knowledge for further retention. This framework is able to provide detailed explanations about the role of long-term memory processes in word learning; on the other hand, it pays little attention to ultra-rapid initial stages of new word forms acquisition, at least some of which have been suggested to bypass the two-stage route and to be instantiated in the neocortex directly. While not refuting the CSL account as such, these studies suggest that it needs a certain revision to account for the rapid neocortical memory-trace formation under FM conditions.

Notably, the vast majority of studies evaluating FM used predominantly behavioral or sluggish hemodynamic measures. While fMRI is an excellent spatially-precise neuroimaging tool, its low temporal resolution makes it less optimal to study rapidly changing neural dynamics underlying language processing and fast plastic changes in brain circuits. Electroencephalography (EEG), on the contrary, can track neuronal electric activity with high temporal precision, which allows scrutinizing neural processes of language learning and comprehension on a millisecond scale. To find electrophysiological correlates of word learning, the most commonly used event-related potential (ERP) component has been the N400, a negative-going event-related brain response linked to lexical and semantic features of verbal stimuli (Kutas and Federmeier, 2011). For instance, N400 dynamics was assessed in healthy adults as they performed a contextual word-learning task in which they were required to derive the meaning of a novel word from a linguistic context provided by a few sentences (Mestres-Missé et al., 2007). After a few exposures to novel words, the N400 amplitude (initially elevated, as is typically the case for nonsense words) became statistically indistinguishable from that elicited by familiar real words, suggesting a rapid neural acquisition effect for the novel items.

Some developmental N400 studies have shown that the ability to quickly map new words and retain those representations develops quite early in childhood (Friedrich and Friederici, 2008). Children as young as 6 months old could quickly associate a new word form with the corresponding referent demonstrated in a picture—word mismatch N400 paradigm (Friedrich and Friederici, 2011). A similar study with 20-month-olds indicated substantial differences in FM efficiency in relation to child’s productive vocabulary size during the period of vocabulary spurt (von Koss Torkildsen et al., 2008): children with high productive vocabularies displayed a significant N400 incongruity effect for violations in word-object mappings.

Importantly, the N400 likely reflects not the process of memory trace activation or word learning *per se*, but rather the integration of the stimulus items (such as old or new lexicon elements) in the broader context of a sentence (Friederici, 2002). On the other hand, earlier ERP components, elicited by single words outside any context in passive auditory exposure, have been shown to directly reflect lexical and lexico-semantic access processes starting from 50 to 150 ms after the auditory information allows for word identification (Shtyrov et al., 2005, 2010; Shtyrov and Pulvermüller, 2007). Several ERP studies in adults using passive perceptual learning paradigms revealed a significant increase of early electrophysiological activity in fronto-temporal cortical networks, indexing rapid learning of novel word forms after a mass exposure (Shtyrov, 2011; Kimppa et al., 2015). Furthermore, the magnitude of this brain response increase for novel word forms was predictive of further recall and recognition of the newly acquired items, supporting the notion that such enhanced neural activity is a genuine neural correlate of the learning process (Kimppa et al., 2015). Similar findings were demonstrated in school-age children using MEG (Partanen et al., 2017), whereas this neurophysiological pattern of rapid memory trace build-up could not be found in children with dyslexia (Kimppa et al., 2018).

While such studies have suggested rapid plastic changes during word acquisition, along with neurophysiological indices of new memory trace build-up, they predominantly did not address the FM mechanism as a single-shot exclusion-based learning. They often used a series of paired word-picture presentations, story-like sentential context, direct explicit instruction or mass repetition. Overall, the neurophysiological underpinnings (and electrophysiological correlates in particular) of FM as a special form of learning still remain largely unexplored. Addressing them was the goal of the present study.

To study the neural correlates of this early implicit learning mechanism in the adult brain, we designed an experimental procedure that could model the process of rapid new word acquisition in a naturalistic FM setting. As an incidental, exclusion-based learning, FM implies inferring a word’s meaning from the existing semantic context *via* *“disjunctive syllogism”* cognitive process (Halberda, 2006; Coutanche and Thompson-Schill, 2014; Atir-Sharon et al., 2015). Thus, we implemented an audio-visual FM learning paradigm that included counterbalanced combinations of familiar and novel words presented auditorily in pseudorandom order in conjunction with novel and familiar images. Similar to the conventional behavioral FM studies, the subject was asked to choose a new object defined by a previously unfamiliar word form, which could only be achieved by excluding other, familiar stimuli. In contrast with the vast majority of previous neuroimaging studies, only a single trial was allowed to carry out this task. This picture-word paradigm was combined with short passive EEG recording sessions run before and after the FM task since passive ERPs are known to reflect memory-trace activation and build-up (Shtyrov, 2012). We hypothesized that rapid formation of word-object associations *via* FM would be indicated by enhanced ERPs dynamics as a result of training exposure. As control conditions, we used, on the one hand, an acoustically similar familiar word undergoing similar single-shot selection task, and, on the other hand, other items that were not subject to FM training.

## Materials and Methods

### Participants

Twelve monolingual native Russian speakers participated in the study [mean age = 23 (*SD* = 3.9); range 18–30 years; five men]. All were right-handed (Edinburgh inventory; Oldfield, 1971) with normal or corrected to normal vision and no record of neurological diseases. All participants were informed about the experimental procedure and signed a consent form. The study was approved by the Ethics Committee of Saint Petersburg State University and conducted in accordance with the Helsinki Declaration.

### Stimuli

#### Acoustic Stimuli

Four acoustically and phonetically similar consonant-vowel-consonant (CVC) triphones were used as stimuli: two of them were meaningful Russian words ([*k^j^it*]—whale and [*kot*]—cat) and the other two were phonologically legal novel word-forms ([*k^j^et*] and [*kat*]). The stimuli were recorded using a monolingual native Russian female speaker and processed in Adobe Audition 3.0 (Adobe Systems Inc., San Jose, CA, USA) and Praat v.6.0.40 (Boersma, 2002) software. Acoustic properties of the stimuli (duration, intensity, F0) were maximally matched, all stimuli were 413 ms in duration.

#### Visual Stimuli

Visual stimuli consisted of two-dimensional pictures of 11 familiar animals and one unknown (unreal) creature taken from the Microsoft Clipart Collection. The mean angular size of the pictures was equal to 3.5°. Acoustic and visual stimuli were presented using the Psytask software (v. 1.41.2; Mitsar Ltd, St. Petersburg, Russia) running on a Windows computer.

### Experimental Design and Procedures

#### Experimental Design

Experimental design included an *FM exposure* and two *passive sessions* that were run immediately before and after the FM. Experiment started with a short *practice session* aimed to familiarize subjects with the task, using other stimuli than those in the main task.

#### Passive Session

Passive sessions were run twice: before and after the FM procedure (which will be described below). During both passive sessions, EEG was continuously recorded. The subjects were seated in a chair facing the computer monitor located 1 m in front of them. They were instructed to pay no attention to the sound stimuli and to watch a silent video film. The acoustic stimuli (two meaningful words and two novel word-forms) were binaurally presented through the headphones at 60 dB SPL. Each stimulus was presented 25 times in pseudorandom order such that the same stimulus was not repeated twice in a row. The stimulus onset asynchrony (SOA) was jittered randomly between 1,000 and 1,100 ms.

To investigate the ERPs dynamics of the word-object association formation, we compared ERPs obtained in response to the novel words between the two passive sessions, i.e., before and after the FM condition. As a control condition, we used, on the one hand, the ERPs obtained in response to the acoustically similar previously familiar word undergoing the similar single-shot selection task, and, on the other hand, two other items (familiar word and unfamiliar pseudo-word) that were not subject to FM training, but were used as a control for the mere repetition of stimuli in the passive session.

#### Fast Mapping

**Figure 1:**
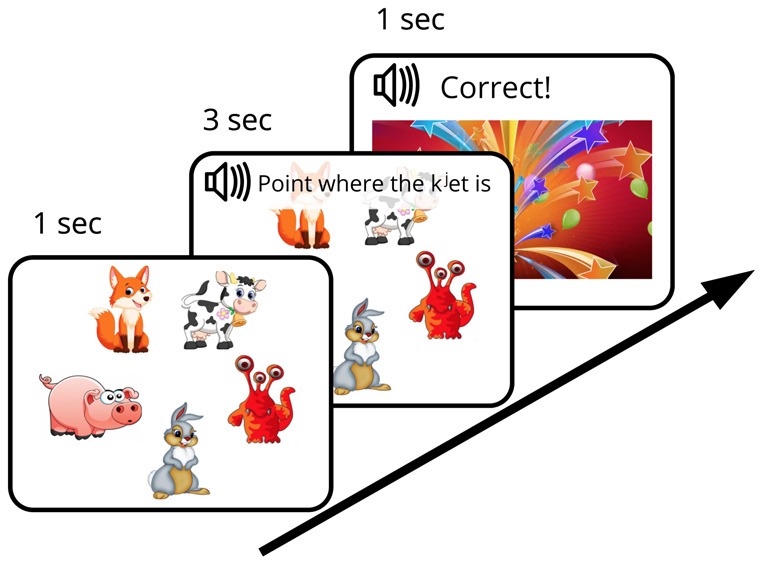
Diagram of fast mapping (FM) session. Experimental design included an *FM exposure* and two *passive sessions* that were run immediately before and after the FM.

We designed an FM procedure with the aim of mimicking the earlier behavioral investigations (such as the original “chromium” study described above) as closely as possible while adapting them to an EEG experiment setting with well-matched control stimuli and strictly defined recognition points allowing for precise ERP time-locking. The designed FM procedure was aimed to investigate the subject’s ability (under the conditions of experimentally created ambiguity) to infer, by exclusion, the referent of a novel word from a brief single exposure and to store this newly formed word-object mapping in memory for later use. To this end, an audio-visual FM paradigm with preferential pointing task was applied (Spiegel and Halberda, 2011). The subject was asked to identify one unknown object among pictures of familiar ones, all presented simultaneously on the screen. First, five objects, arranged in a circle and counterbalanced for position, appeared on the screen on a white background. After a short delay (~1 s) an auditory request (3 s long) relating to one of the objects was made (“Point, where X is”) and the subject had to identify which object was being referred to (in case of the novel word, this was only possible by excluding other, familiar, objects) and to point to it. The FM session started with a short *practice session* that included two trials with familiar word-object combinations [e.g., [*gus*^j^] (goose), [*kon^j^*] (horse)], which were not used in the experiment proper. After that, one trial with familiar word [*kot*] (cat) paired with a known visual item and one FM trial with the target new word form ([*k^j^*et]) paired with an image of the novel visual item, displayed together with four familiar objects, were presented (Figure 1). After the subjects succeeded in referent selection (familiar or target new word), they were greeted by the experimenter and a colorful picture of firework appeared on the screen (for 1 s). Whereas one real word and one novel word form from the set of four underwent this FM procedure, the other two items (acoustically similar real word and pseudo word) were used as control stimuli, i.e., they were present in the passive ERP recordings but not in the FM condition. The small number of stimuli was used in order to both approximate the early behavioral designs, which typically used a single novel item in an exposure session, and to avoid any potential interference that could arise should multiple items be used.

### Data Analysis

EEG was continuously recorded using a 32-channel Mitsar EEG set-up and WinEEG software (Mitsar Ltd) with 500 Hz sampling rate and pass-band of 0.01–150 Hz. Ag/AgCl electrodes were mounted in an extended 10-20-system electrode cap; hardware-linked earlobe electrodes were used as the reference channel. To control for eye-movements, horizontal and vertical electro-oculograms (EOG) were recorded. The impedance of the electrodes did not exceed 10 kOhm. The signal was bandpass filtered between 0.5 and 45 Hz offline.

EEG data obtained during the passive sessions were epoched from 100 ms before to 700 ms after the stimulus onset. The baseline was corrected using a 100-ms pre-stimulus interval. EEG epochs in which the EEG or EOG signal amplitude exceeded ±100 μV on any of the electrodes were omitted. The average number of trials remaining after artifact removal was 21.4 ± SD 2.5 out of the total of 25 per type. Two subjects were excluded from the final dataset due to excessive artifacts in the EEG recordings; thus, 10 subjects were included instatistical analysis.

Amplitude analysis was carried out for the fronto-central electrode cluster where the auditory evoked responses are typically maximal: F3, Fz, F4, FC3, FCz, FC4, C3, Cz, and C4. Visual data inspection revealed the presence of several pronounced peaks in a broad 100–500 ms time window. Since we were agnostic with respect to the latency when FM effects might occur, we opted for an unbiased data-driven approach and split the epochs into equal 100-ms bins, and performed an exploratory analysis of each of the four bins. Mean ERP amplitudes were calculated over 100 ms time intervals, starting from 100 ms, when the stimuli could be differentiated acoustically. These were analyzed for each stimulus type separately using the repeated measures analysis of variance (rmANOVA; SPSS v. 21, IBM Corporation, New York, NY, USA) with *Session* (before/after FM), *Electrode* (frontal, central-frontal, central) and *Location* (left, right, medial) factors. Greenhouse-Geisser correction was applied whenever the sphericity assumption was violated; multiple comparisons were corrected for using Bonferroni corrections where necessary. Average ERPs for each stimulus type were calculated by combining epochs of each familiar or novel word separately. Effect sizes were calculated using partial eta squared ($\eta_{\text{p}}^{2}$; SPSS v. 21, IBM Corporation, New York, NY, USA).

Low-resolution electromagnetic tomography (LORETA; Pascual-Marqui et al., 1994) images were obtained by estimating the current source density distribution of brain electric activity on a dense grid of 2,394 voxels at 7-mm spatial resolution applied to the digitized Talairach human atlas (Talairach and Tournoux, 1988). To this end, the group-average difference between the ERPs recorded before and after FM session was submitted to LORETA. Group-average data were used since they benefit from a much-increased signal-to-noise ratio that source analysis algorithms are highly sensitive to, which, in turn, could somewhat compensate for the low resolution of the EEG technique applied.

## Results

The FM session was completed successfully by all subjects. Here, we present the results of comparing the ERP data collected in passive sessions run before and after the FM learning condition. ERPs were recorded to passively presented auditory stimuli, including familiar and novel items used in the FM session and untrained control stimuli.

We split the epochs into equal 100-ms bins during the time when most typical word-related ERPs might take place and performed an exploratory analysis of each of the 4 bins. Analysis of ERP data using rmANOVA is presented in Table 1. Analysis of data from the 100–200 ms window (which showed a negative peak in the subtraction curve with 155 ms average latency) revealed no significant main effects of *Session, Electrode* or *Location* as well as no interaction effects.

**Table 1:** Analysis of variance (ANOVA) results.

SM	100–200 ms	200–300 ms	300–400 ms	400–500 ms
Session	*F*_(1,9)_ = 0.545	*F*_(1,9)_ = 5.398	*F*_(1,9)_ = 8.428	*F*_(1,9)_ = 0.995
	*p* = 0.479	*p* = 0.045*	*p* = 0.018*	*p* = 0.345
	*$\eta_{\text{p}}^{2}$* = 0.057	*$\eta_{\text{p}}^{2}$* = 0.375	*$\eta_{\text{p}}^{2}$* = 0.484	*$\eta_{\text{p}}^{2}$* = 0.100
Electrode	*F*_(1.319,11.875)_ = 1.542	*F*_(1.302,11.716)_ = 0.189	*F*_(1.122,10.099)_ = 3.167	*F*_(1.129,10.162)_ = 2.351
	*p* = 0.247	*p* = 0.736	*p* = 0.103	*p* = 0.155
	*$\eta_{\text{p}}^{2}$* = 0.146	*$\eta_{\text{p}}^{2}$* = 0.021	*$\eta_{\text{p}}^{2}$* = 0.260	*$\eta_{\text{p}}^{2}$* = 0.207
Location	*F*_(1.781,16.025)_ = 0.320	*F*_(1.736,15.627)_ = 0.434	*F*_(1.691,15.222)_ = 3.407	*F*_(1.756,15.800)_ = 7.827
	*p* = 0.706	*p* = 0.628	*p* = 0.066	*p* = 0.005*
	*$\eta_{\text{p}}^{2}$* = 0.034	*$\eta_{\text{p}}^{2}$* = 0.046	*$\eta_{\text{p}}^{2}$* = 0.275	*$\eta_{\text{p}}^{2}$* = 0.465

Analysis included data from nine electrodes, with factors of Session, Electrode and Location. No significant main effects of interaction were revealed over all selected time windows. Significant results (*p* < 0.05) are highlighted by an asterisk (*).

**Figure 2:**
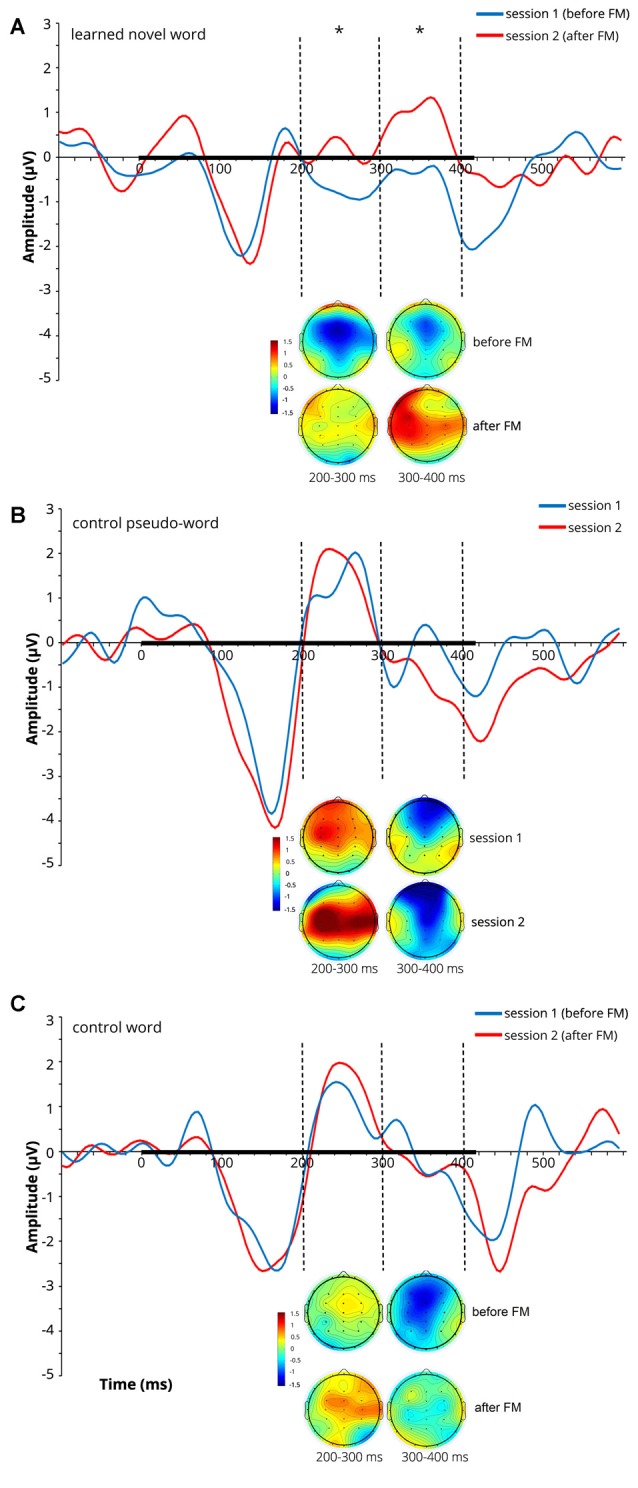
Average event-related potentials (ERPs) at Cz and mean voltage topographic scalp maps before and after FM condition for the learnt novel word **(A)**, control pseudo-word **(B)** and control familiar word **(C)**. Dotted lines indicate 200–300 and 300–400 ms windows, where significant effects were found in the FM condition. Black bar on the x-axis shows the stimulus duration. Scalp topography maps show the amplitude distribution averaged over selected time windows. Asterisks denote statistical significance: *p < 0.05. Displayed data bandpass-filter 1–20 Hz, for illustration purposes only.

At later latencies, a significant main effect of *Session* on the ERP amplitude was found over both the 200–300 ms (*F*_(1,9)_ = 5.398, *p* = 0.045, $\eta_{\text{p}}^{2}$ = 0.375) and 300–400 ms (*F*_(1,9)_ = 8.428, *p* = 0.018, $\eta_{\text{p}}^{2}$ = 0.484) windows for the learnt novel word form reflecting an amplitude increase after the FM condition (note that both windows showed positive-going peaks in the subtraction curves at 250 ms and 360 ms). No significant main effects of *Electrode* and *Location* and no interaction effects were found in those time windows. Average ERPs at Cz and mean voltage topographic scalp maps before and after FM condition for the target novel word form are shown in Figure 2A.

A significant effect of *Location* (*F*_(1.756,15.800)_ = 7.827, *p* = 0.005, $\eta_{\text{p}}^{2}$ = 0.465) was found over the 400–500 ms window. Multiple pair-wise comparisons revealed the significant amplitude enhancement for the novel and familiar word forms in the left hemisphere as compared to the right hemisphere (*p* = 0.004). No significant main effects of *Session* and *Electrode* and no interaction effects were found in this time interval.

**Figure 3:**
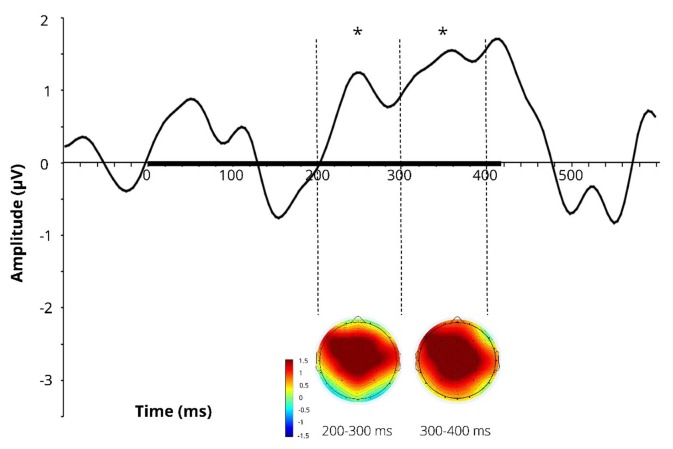
Difference wave (at Cz) obtained by subtracting the ERPs for the learnt novel word used in passive session 1 (before FM) from those in passive session 2 (after FM) and corresponding difference topographic scalp maps for the learnt novel word. Dotted lines indicate 200–300 and 300–400 ms windows, where significant effects were found in the FM condition. Black bar on the x-axis shows the stimulus duration. Asterisks denote statistical significance: *p < 0.05. Displayed data bandpass-filter 1–20 Hz, for illustration purposes only.

Difference wave (at Cz) obtained by subtracting the ERPs for the learnt novel word used in passive session 1 (before FM) from those in passive session 2 (after FM) and corresponding difference topographic scalp maps are presented in Figure 3. Additional figures for control pseudoword and control familiar word are presented in Supplementary Figure S1.

In addition, the analysis of N400 component indicated that the N400 peaked at 416 ± 36 ms in the before-FM condition. Thus, we conducted an *ad hoc* analysis of N400 component in the a 100-ms window centered on this peak (i.e., 366–466 ms), which confirmed the significant *Location* main effect (*F*_(1.878,16.898)_ = 10.116, *p* = 0.001, $\eta_{\text{p}}^{2}$ = 0.529) that was obtained at 400–500 ms time bin. However, no *Session* effect was observed (*F*_(1,9)_ = 3.938, *p* = 0.078, $\eta_{\text{p}}^{2}$ = 0.304).

**Figure 4:**
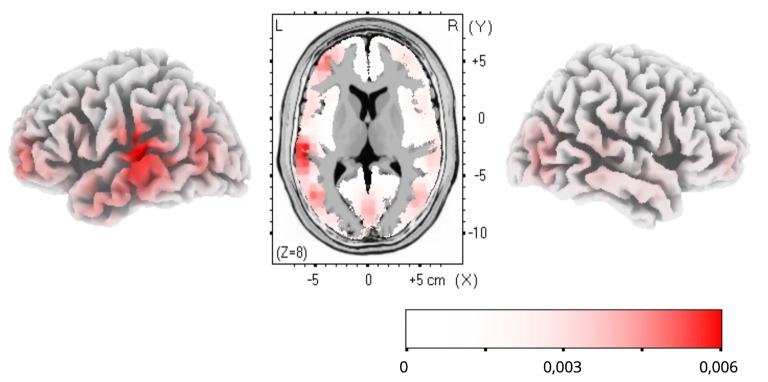
Low-resolution electromagnetic tomography (LORETA) source estimation of neuronal activity changes after the novel word learning exposure (post- minus pre-FM contrast) in the 200–300 ms time window.

To estimate cortical sources of the training-related ERP dynamics, LORETA computation in Talairach space was applied to group-average subtractions of ERP traces before and after FM condition in the time windows of significant ERP effects. The LORETA results are shown in Figure 4. Maximal activity was observed in the left temporal cortex (peaking in BA21), with a less pronounced source in the left anterior prefrontal cortex. No differences were found for the control familiar word used in the FM condition or control items given in passive sessions only (Figures 2B,C).

## Discussion

This study aimed at delineating neural correlates of FM of phonologically and semantically novel words through a single-shot exposure in a naturalistic inference-based learning scenario. We found a significant enhancement in ERP amplitudes elicited by a native novel word form following this simple semantic learning task. This enhancement was found using passive auditory ERPs, known to be an index of automatic memory trace activation (Shtyrov et al., 2005, 2010; Shtyrov and Pulvermüller, 2007), and was maximal over 200–400 ms after the word onset, i.e., shortly after the words could be identified as distinct and even before their offset. Notably, no difference was found for either the native familiar words used in the same experimental conditions or for the control phonologically legal pseudoword given in passive sessions only. These different types of control conditions rule out a possibility that the current ERP dynamics could simply be explained based on physical stimulus repetition; instead, the observed change in the brain’s response seems to be best interpreted as a specific consequence of the FM procedure.

Previous studies have shown that formation of neural memory traces for novel spoken word forms with native phonology could be captured after a mass exposure, with multiple (sometimes dozens or hundreds) repetitions (Shtyrov, 2011; Kimppa et al., 2015; Partanen et al., 2017) whereas the current rapid ERP dynamics was revealed after a one-trial exposure to new word-picture pairs. Given the control conditions employed (involving FM word as well as non-FM word and pseudoword presented in the passive session only), the present result points toward semantic context advantage in the rapid formation of novel memory traces for words.

These results are similar to some previous investigations that found online changes of brain dynamics in the process of novel meaning acquisition implemented through word-picture associations or sentential context (e.g., Breitenstein et al., 2005; Mestres-Missé et al., 2007). Still, to our knowledge, the current data is the first electrophysiological *evidence of FM proper, as the process of exclusion-based inference learning implemented in a single shot*. While previous studies focused on using purely behavioral or slow hemodynamic measures, here, this neurophysiological signature of the processes underlying rapid word acquisition in healthy adults is documented as a dynamic enhancement of electrophysiological response. This enhancement is most likely underpinned by an automatic activation of the newly created word memory trace, realized as a robust neuronal circuit formed in the process of associative learning (Pulvermüller et al., 2001; Aleksandrov et al., 2011).

Some previous studies (Wilding, 1999, 2000) recorded ERPs during recognition memory tasks aimed to differentiate old (studied) and new (untrained) visually presented words. ERPs to words judged correctly to be old were more positive (at left parietal sites) than new ones. This left-lateralized old/new effect is known to index the process of recollection (retrieval) from episodic memory. Moreover, its magnitude appeared to be related to the amount/quality of information retrieved from memory. Our findings are somewhat similar in the amplitude patterns, even though our study was designed with a different paradigm and assessment technique: in conjunction with FM paradigm (not aimed at memorizing as such, but rather at incidental learning through inference), we used passive auditory ERPs that are known to be a neurophysiological index of automatic memory trace activation and build-up (Shtyrov, 2012; Kimppa et al., 2015; Partanen et al., 2017) rather than active retrieval. Notably, the magnitude of this brain response increase for novel word forms is predictive of further recall and recognition of the newly acquired items (supporting the notion that such enhanced neural activity is a genuine neural correlate of the learning process, Kimppa et al., 2015), which again bears clear similarity to the Wilding’s studies above.

The topographic analysis of the amplitude distribution of these ERP changes suggested a more pronounced left laterality effect of FM on novel word-form learning. Additionally, analysis of cortical activity sources using LORETA confirmed that the learning dynamics was underpinned by sources in the left temporal and inferior-frontal cortices, indicating that this response enhancement is likely underpinned by the perisylvian neural network specialized in native language processing. Overall, it may be proposed that FM may induce rapid neocortical plasticity in healthy adult brain by engaging pre-existing language neural networks for mastering new word forms with native phonology (Shtyrov, 2011; Kimppa et al., 2015; Partanen et al., 2017). On a more cautious note, since, for increased SNR which source-analysis algorithms are highly sensitive to, grand-average data were used for LORETA estimations, no statistical verification of the source activation is possible and the results should, therefore, be treated as indicative of an average “center of gravity” of cortical generators, rather than definitive. That implies that the present source analysis outcomes should be treated with extreme caution and must be verified in future studies (using, e.g., combined EEG/MEG with MR-based cortical models) with respect to the exact effect origins. That said, the left temporo-frontal distribution of the LORETA effects found is well compatible with existing knowledge of the cortical language and learning systems and thus still provides a useful illustration of putative neural underpinnings of the FM mechanism.

ERP dynamics observed here exhibited some differences from the earlier investigations. First, in our study the earliest registered activity manifesting differential dynamics after semantic learning task was around 200–400 ms with a left-central distribution of positive polarity. Several previous studies demonstrated earlier rapid lexical effects in auditory ERPs starting from already ~50–100 ms with predominantly negative polarity deflections and less lateralized fronto-central distribution (e.g., Shtyrov et al., 2010). Those studies, however, predominantly time-locked ERPs to word-recognition points located at word offsets, whereas here the word identification became possible in the initial CV transition; while it is more difficult to precisely indicate these transition points, they likely occur within the first ~200 ms after onset, implying that, in terms of word recognition, our latencies are comparable with previous studies. Interestingly, no significant *Session* effect was observed in the N400 time window. A potential explanation for this is that the typical N400 effects reflect the integration of single words into wider (sentential) context. Here, however, only single words were presented outside any phrases, and the putative lexico-semantic activation took place at an earlier time interval rather than in the typical N400 range. As for polarity, at least one earlier ERP experiment reported an increase in frontal positivity during rapid language learning (Shtyrov, 2011), which is what we found here as well, even though we implemented a rather different learning paradigm and stimuli. Finally, previous fMRI work suggested ATL as the primary hub for implicit FM learning (e.g., Merhav et al., 2015), whereas our LORETA results suggest a more posterior-superior temporal lobe activation; this divergence, however, cannot be resolved based on the current data: on the one hand, activation in the temporal pole is known to be unreliable in both fMRI and EEG; on the other hand, the present source reconstruction results should be treated with extreme caution as they are based on low-resolution EEG data and present a group-average picture which cannot be verified statistically. Further investigations of the exact learning-related neural dynamics and of their neuroanatomical origins are clearly needed to scrutinize these processes in more detail; one way to pursue this could be to use combined MEG/EEG with individual MR-based source reconstructions techniques.

Notably, the very brief and subject-friendly novel paradigm we have developed—based on a non-demanding single-shot learning task and short passive auditory ERP recording—allows for investigations of FM processes in diverse populations, including young children, elderly subjects or different patient groups. Future studies may apply and further develop this approach to assess learning-related neural dynamics and their deficits in different conditions, populations and experimental settings.

In this experiment, we have followed the strategy of the original behavioral FM studies that typically used a single novel item in one exposure session. The logic for this is two-fold. First, it was aimed at mimicking the earlier investigations as closely as possible, while adapting them to an EEG experiment setting with two types of control stimuli and strictly defined recognition points, to allow for precise ERP time-locking. Second, to our knowledge, no previous study has electrophysiologically investigated single-shot learning in classical FM situation in its strict sense; therefore, in this first endeavor, we opted for a safe approach avoiding any potential interference from using multiple items. Previous EEG studies did not address the FM mechanism in its strict sense as a one-trial inference-based learning; instead, they used series of paired word-picture presentations of the same items, story-like sentential context with multiple item occurrences or even mass stimulus repetition. In contrast to the vast majority of such previous behavioral and neuroimaging studies, in our study, only a single trial was allowed to carry out the FM task. This strategy has obviously proved to be fruitful in the present case. Indeed, on the one hand, we found a significant enhancement in ERPs amplitudes after a one-trial exposure to the newly inferred item, which appears to be an important advance on its own. On the other hand, however, such a restricted stimulus design does not easily allow for general conclusions concerning the current findings and the neurobiological mechanisms involved. Therefore, taken at face value, the current result should still be treated with caution. We suggest that future studies should expand the approach developed here to possibly use several FM items to both validate our results and generalize them.

On a similarly cautious note, even though the effect sizes obtained here are fairly good and results clearly demonstrate significant ERP changes following the FM procedure, we suggest that, for reliability and reproducibility, future studies could use larger subject samples than the that employed here. A somewhat more difficult question relates to the number of trials employed in the present passive sequence. Considering that the massive stimulus repetition *per se*, as discussed above, leads to memory trace build-up even in passive designs without any semantic training, we limited the passive sessions to 25 trials only. This number is on the low end of scale for a reliable ERP (although this *per se* does not undermine the present result); future studies could circumvent this issue by using multiple tokens (e.g., 2–4, with 25 repetitions each) that can be combined to produce ERPs with higher SNRs.

In sum, the results of the current study suggest that the FM mechanism of word acquisition, well established in previous behavioral research, promotes incidental rapid integration of new associations into neocortical lexico-semantic networks in healthy adult brain as indicated by the rapid changes in ERPs present after a brief single exposure to a novel item. Future studies are needed to validate the current findings and generalize them to other stimulus types, languages and experimental groups, to clarify the neuroanatomical underpinnings of this mechanism as well as to scrutinize these neural FM processes in typical and atypical development.

## Data Availability

The raw data supporting the conclusions of this manuscript will be made available by the authors, without undue reservation, to any qualified researcher.

## Ethics Statement

The study was approved by the Ethics Committee of Saint Petersburg State University and was conducted in accordance with the Declaration of Helsinki. All subjects gave written informed consent.

## Author Contributions

AA and YS designed the study. MV and VK performed the experiment and data analysis. YS supervised this work. All authors discussed the results and contributed to the final manuscript.

## Conflict of Interest Statement

The authors declare that the research was conducted in the absence of any commercial or financial relationships that could be construed as a potential conflict of interest.

## Supplementary Material

FIGURE S1 Difference wave (at Cz) obtained by subtracting the ERPs for the learnt novel word used in passive session 1 (before FM) from those in passive session 2 (after FM) and corresponding difference topographic scalp maps for the learnt novel word **(A)**, control pseudo-word **(B)** and control familiar word **(C)**. Dotted lines indicate 200–300 and 300–400 ms windows, where significant effects were found in the FM condition. Black bar on the x-axis shows the stimulus duration. Asterisks denote statistical significance: **p* < 0.05. Displayed data bandpass-filtered at 1–20 Hz, for illustration purposes only.
